# Immediate response of paddy soil microbial community and structure to moisture changes and nitrogen fertilizer application

**DOI:** 10.3389/fmicb.2023.1130298

**Published:** 2023-07-21

**Authors:** Linrong Han, Hongling Qin, Jingyuan Wang, Dongliang Yao, Leyan Zhang, Jiahua Guo, Baoli Zhu

**Affiliations:** ^1^Shaanxi Key Laboratory of Earth Surface System and Environmental Carrying Capacity, College of Urban and Environmental Sciences, Northwest University, Xi’an, China; ^2^Key Laboratory of Agro-Ecological Processes in Subtropical Region, Institute of Subtropical Agriculture, Chinese Academy of Sciences, Changsha, China; ^3^College of Resources and Environment, Hunan Agricultural University, Changsha, China; ^4^College of Biodiversity, Conservation Southwest Forestry University, Kunming, China

**Keywords:** paddy soil, fertilization, moisture levels, microbial community, AOA, AOB, network analysis

## Abstract

Water and fertilizer managements are the most common practices to maximize crop yields, and their long-term impact on soil microbial communities has been extensively studied. However, the initial response of microbes to fertilization and soil moisture changes remains unclear. In this study, the immediate effects of nitrogen (N)-fertilizer application and moisture levels on microbial community of paddy soils were investigated through controlled incubation experiments. Amplicon sequencing results revealed that moisture had a stronger influence on the abundance and community composition of total soil bacteria, as well as ammonia oxidizing-archaea (AOA) and -bacteria (AOB). Conversely, fertilizer application noticeably reduced the connectivity and complexity of the total bacteria network, and increasing moisture slightly exacerbated these effects. NH_4_^+^-N content emerged as a significant driving force for changes in the structure of the total bacteria and AOB communities, while NO_3_^−^-N content played more important role in driving shifts in AOA composition. These findings indicate that the initial responses of microbial communities, including abundance and composition, and network differ under water and fertilizer managements. By providing a snapshot of microbial community structure following short-term N-fertilizer and water treatments, this study contributes to a better understanding of how soil microbes respond to long-term agriculture managements.

## 1. Introduction

Water and nutrients availability are two critical factors that limit global crop productivity. Chemical fertilizer application has been one of the most important practices of modern agriculture (Soman et al., 2017; Chen et al., 2019; Li G. C. et al., 2021). Fertilization enhances soil fertility by increasing nutrient levels. However, current estimates suggest that the efficiency of fertilizer use is relatively low, with only 40%–50% of the applied nitrogen (N)-fertilizer being utilized by crops (Bijay-Singh and Craswell, 2021). Overfertilization not only depletes resources but also gives rise to various environmental issues, including water pollution, soil degradation, greenhouse gas emissions, and loss of biodiversity (Francioli et al., 2016).

Microorganisms, as an integral component of the soil ecosystem, play a crucial role in maintaining soil quality and functions, and they are highly sensitive to changes in soil conditions (Li et al., 2016; Pan et al., 2016; Chen et al., 2017; Banerjee et al., 2018). Microorganisms drive the biogeochemical cycles of soil carbon and nitrogen, thus regulating the size of the soil carbon pool and the emissions of greenhouse gases like methane and N_2_O into the atmosphere (Chai et al., 2019). Different agriculture management practices influence soil microbial communities through various mechanisms (Liang et al., 2012). For instance, manure application improves soil texture and organic matter content, thereby stimulating the growth and activity of soil microorganisms (Gomiero et al., 2011; Tang et al., 2019). In addition to benefiting crop yield, fertilization also provides nutrients and substrates for soil microbes (Feng et al., 2015; Yu et al., 2019). Soil moisture directly or indirectly affects microbial activities by regulating oxygen concentration and nutrient availability (Drenovsky et al., 2004; Pan et al., 2016). Furthermore, the presence of free water connecting soil particles can facilitate the mobility of nutrient and microbial cell, thereby shaping the structure of the soil microbial community (Zhou et al., 2002; Yuan et al., 2016).

Both fertilizer application and water content play crucial roles in sustaining crop productivity. However, their impact on soil microbial community structure and associated ecosystem function is not always positive. For instance, the addition of fertilizer can stimulate microbial activity, leading to the decomposition of organic matter and denitrification, which can result in soil carbon loss and the emission of greenhouse gases (Canarini et al., 2016; Bastida et al., 2017; Tian et al., 2017).

Moisture levels and the availability of N-compounds are considered important factors in regulating the communities of nitrifying and denitrifying microbes (Qin et al., 2021; Liu et al., 2022), whose activities determine soil N_2_O emission fluxes (Wang et al., 2019; Jia et al., 2021). Interestingly, the highest N_2_O emissions are often observed at different moisture levels, suggesting that microbial communities in diverse environments respond differently to the same factor. This response is influenced by synergistic interactions with other elements such as soil type, vegetation and historical conditions (e.g., Liu et al., 2018; Qin et al., 2020).

Contrasting responses in nitrification rates and denitrification enzyme activities to moisture and N-fertilizer addition have been reported in grassland soils from China and Australia (Long et al., 2017). Additionally, a microcosm simulation study highlighted the legacy effects of soil moisture on microbial community structure and the transcription of genes encoding key enzymes involved in N-cycling (Banerjee et al., 2016). In the Three Gorges Reservoir environment, ammonia oxidizing archaea (AOA) have been suggested to be more adaptive than ammonia oxidation bacteria (AOB) to water level fluctuations, while AOB have shown greater competitiveness than AOA in riparian soils of the Miyun Reservoir in Beijing (Wang et al., 2016; Liu et al., 2018).

The effects of agricultural practices on soil microbial communities and their activities are intricate, often requiring long-term implementation to observe significant changes. For instance, changes in microbial diversity have been captured after decades of organic framing (Hartmann et al., 2015) or reduced tillage (Tyler, 2019). However, microbial community changes are dynamic and exhibit spatiotemporal heterogeneity. Therefore, snapshots taken after long-term treatments provide limited insight into how microbes initiate their responses and adapt to these conditions. Additionally, the initial response of microbial communities to specific soil conditions remains unclear.

In this study, we aimed to investigate the responses of soil microbial communities and key ammonia-oxidizing microbes to short-term changes in moisture levels, represented by different water filled pore space (WFPS) values, and nitrogen fertilization, through high-throughput 16S rRNA and *amoA* genes sequencing. The abundance, community structure and network connections of total bacteria and ammonia-oxidizing microbes were scrutinized to address two primary objectives: (1) understanding how the soil bacterial community responds to short-term N-fertilization and water treatments, and (2) examining the combined effects of moisture levels and fertilization on the indigenous microbial community.

## 2. Materials and methods

### 2.1. Soil sample preparation

Paddy soil samples were collected from the National Agroecological Research Station (111°26′ E, 28°55′ N, altitude: 92.2–125.3 m) in Taoyuan, Hunan Province, China. The region is characterized by a subtropical humid monsoon climate, with an average annual temperature of 16.5°C, an average annual precipitation of 1,448 mm, an average daily sunshine duration of 15 h and 13 min, and an annual frost-free period of 283 days. Soil samples (0–20 cm) were collected, sieved (<2 mm), and stored. The soil is silty clay, developed from red clay, comprising 31.1% clay (<0.002 mm), 53.0% silt (0.002–0.05 mm), and 15.9% sand (0.05–2.00 mm). The other main soil properties were as follows: pH, 5.06; Soil Organic Matter, 34.73 g kg^−1^; Total Phosphorus, 0.66 g kg^−1^; Total N, 2.22 g kg^−1^; Total Potassium, 11.76 g kg^−1^ (Qin et al., 2020).

### 2.2. Experimental design

In order to initiate the activity of microorganisms, the soil was pre-incubated for 2 days under dark conditions at 25°C, with 25% WFPS. Then microcosm culture experiments were set up in 1000 mL glass jars, each with 200 g (dry weight) preconditioned soil and was covered with a film to facilitate gas exchange. Two fertilization conditions, with nitrogen-fertilizer (NF) and without (CK) were prepared. For the NF treatment, NH_4_NO_3_ was applied at 720 μg N/g dry soil, which was equivalent to 200 kg N/ha on a surface area basis (200 g soils possess a surface area of 72 cm^2^). For each treatment, five soil moisture levels, namely, 25%, 50%, 75%, 100%, and 125% WFPS were maintained, as previously described (Qin et al., 2020). All microcosms were incubated at 25°C for 96 h.

### 2.3. DNA extraction

At the end of incubation, 0.5 g of soil samples were used for DNA extraction as previously described (Qin et al., 2020), DNA quality and concentration were measured using a spectrophotometer (NanoDrop ND-1000; ThermoFisher Scientific, Germany). For each incubation, three extractions were performed, and the DNA were pooled and stored at −80°C for further analysis.

### 2.4. PCR amplification

The AOA *amoA* gene, AOB *amoA* gene, and 16S rRNA gene sequences were amplified from soil DNA by PCR. A 25 μL PCR reaction contained 30 mM Tris-HCl (pH 8.3), 50 mM potassium chloride, 1.5 mM magnesium chloride, 10 μg bovine serum albumin, 200 μM of each deoxyribonucleoside triphosphate, 1.5 U of Taq DNA polymerase, 25 ng soil DNA and respective primers. Deionized water instead of DNA was used as a negative control. AOA, AOB *amoA* genes were amplified using Arch-amoA 23F (5′-ATGGTCTGGCTWAGACG-3′) and Arch-amoA 616R (5′-GGGGTTTCTACTGGTGGT-3′); amoA-1F (5′-GGGG TTTCTACTGGTGGT-3′) and amoA-2R (5′-CCCCTCKGSAAAGCCTTCTTC-3′), respectively; and for 16S rRNA the primer pair 1369F (5′-CGGTGAATACGTTCYCGG-3′) and 1492R (5′-GGWTACCTTGTTACGACT-3′) was used (Sahan and Muyzer, 2008). The amplification conditions for amoA genes were as follows: 95°C for 5 min, followed by 40 cycles: 94°C for 45 s, 60°C for 1 min, 72°C for 1 min; and a final extension at 72°C for 10 min. The PCR procedure for 16S rRNA was the same, except the annealing temperature was 54°C.

### 2.5. qPCR

qPCR was performed using ABI7900HT (Applied Biosystems, Foster City, CA, United States). qPCR was performed by the SYBR Green method. 16S rRNA-1369F/1492R was used to quantify total bacteria abundance, using Arch-*amoA* 23F/A616R and amoA-1F/2R for AOA and AOB, respectively (Rotthauwe et al., 1997; Tourna et al., 2008). Plasmids containing the target fragments diluted in 10 x series were used to make qPCR standard curves. The reaction systems are all 10 μ L, including:5 μL 2× SYBR green mix II (TaKaRa Biotechnology Co. Ltd., Dalian, China), 1 μL (10 μM) of forward and reverse primers, 0.2 μL 50× Rox Reference Dye (TaKaRa Biotechnology Co. Ltd., Dalian, China), 5 ng DNA template and deionized water.

The 16S rRNA qPCR procedure was as follows: 95°C pre-denaturation for 30 s; followed by 40 cycles of 95°C denaturation for 5 s, 60°C annealing for 30 s, 72°C extension for 30 s; and then a final extension at 72°C for 1 min. The AOA and AOB *amoA* gene qPCR procedures were the same, except that the annealing temperature was 53°C and 55°C, respectively. Deionized water instead of soil DNA was used as a negative control to determine DNA contamination (Vestergaard et al., 2017). Three parallels were done for each sample. The results were required to have an amplification efficiency greater than 95%, R^2^ > 0.999, and a single peak for the melting curve.

### 2.6. AOA, AOB *amoA* gene and total 16S rRNA amplicon sequencing

The PCR processing, sequencing, and analysis of Illumina MiSeq sequencing data were performed as described in Qin et al. (2020). PCR mixtures contained 4 μL of 5× TransStart FastPfu buffer, 0.8 μL of forward primer (5 μM), 0.8 μL of reverse primer (5 μM), 2 μL of 2.5 mM dNTPs, 10 ng of template DNA and filled up to 20 μL with ddH_2_O (TransGen, Beijing, China). PCR products were purified using AxyPrep DNA purification kit (Axygen Bio, United States), and were pooled in equimolar. Paired-end sequencing was performed on the Illumina Miseq platform (Illumina, San Diego, United States) at Shanghai Majorbio BioPharm Technology Co., Ltd., Shanghai. Raw FASTQ files were demultiplexed and quality filtered using QIIME2-2021.4. Barcodes were trimmed, low quality and chimeric sequences were deleted. All samples were normalized to a similar sequencing depth using MOTHUR. Operational clustering of taxonomic units (OTUs) was performed using UPARSE v7.1, based on a cut-off of 97% similarity (Yang et al., 2018). Taxonomic classification was using RDP and FunGene-amoA database for 16S rRNA and amoA sequences, respectively. Representative sequences of each OTU were analyzed using NCBI BLAST to further validate their taxonomy.

### 2.7. Network analysis

Co-occurrence network analyses were performed to explore how nitrogen fertilization and moisture treatments affect the co-occurrence patterns of microbial communities. Interaction networks were constructed using CoNet v1.1.1 in Cytoscape v.3.6.1 based on the Pearson and Spearman correlation values, mutual information similarity, and Bray–Curtis and Kullback–Leibler dissimilarity measures. All networks were visualized using the Fruchterman–Reingold layout with 9,999 permutations and implemented in Gephi. Global network properties such as average path length, average clustering coefficient, and positive and negative correlation of links are calculated.

### 2.8. Statistical analysis

Analysis of variance (ANOVA) was run in SPSS v. 18.0 and used to test the significant effects of moisture and fertilization treatments on soil physiochemical properties, microbial gene abundance and diversity. The copy numbers of all functional genes were log-transformed, and the normality of all data was checked before ANOVA. Principal co-ordinates analysis (PCoA) was used to assess the similarities between the community composition of AOA, AOB and total bacteria. Redundancy analysis (RDA) was used to evaluate the effect of soil properties (exchangeable NH_4_^+^-N, NO_3_^−^-N, and DOC) on the community composition of AOA, AOB and total bacteria. ANOSIM (analysis of similarities) based on the Bray–Curtis distances of OTUs was used to measure the effects of moisture, fertilization, and their interactions on the community composition of AOA, AOB and total bacteria. PCoA, RDA and ANOSIM analysis were performed using R statistical software.

## 3. Results

### 3.1. Soil characteristics

N-fertilizer application clearly increased inorganic N content in the NF treatments, in which nitrate concentrations significantly dropped with the increasing WFPS, and was undetectable in the 125% WFPS incubations, indicating a higher nitrate-consuming activity in higher moisture soils. Nitrate concentration was very low in all CK incubations, regardless the WFPS levels. Ammonium concentration peaked at moderate moisture level of 75% and 50% WFPS for the CK and NF incubations, respectively. However, if treating ammonium in the CK incubations as indigenous background and deducting it from corresponding NF incubations, the remaining ammonium (net NH_4_^+^) in NF incubations showed a decreasing trend along increasing WFPS levels, suggesting a possible active net ammonium consumption under higher moisture conditions (Table 1). DOC level in both treatments was similar under low moisture condition (25% and 50% WFPS), and it significantly increased when moisture was high (100% and 125% WFPS), especially in these of the CK treatment (Table 1).

**Table 1:** Physicochemical properties of soil under different WFPS and fertilization treatments.

WFPS	NO_3_^−^-N (mg/kg)		NH_4_^+^-N (mg/kg)		DOC (mg/kg)	
	CK	NF	CK	NF	CK	NF
25%	0.85 ± 0.17_a_	343.65 ± 42.23_a_	74.43 ± 2.93_e_	388.71 ± 26.48_bc_	350.22 ± 32.18_b_	317.32 ± 30.5_b_
50%	0.49 ± 0.03_b_	324.74 ± 12.13_a_	135.53 ± 2.72_b_	432.14 ± 11.27_a_	311.2 ± 20.9_b_	293.79 ± 26.38_b_
75%	─	131.96 ± 62.42_b_	142.93 ± 3.11_a_	410.97 ± 13.41_ab_	334.23 ± 20.92_b_	405.42 ± 44.45_a_
100%	─	20.85 ± 29.6_c_	127.05 ± 4.94_c_	372.79 ± 35.81_cd_	624.04 ± 17.32_a_	458.62 ± 53.18_a_
125%	─	─	96.05 ± 6.34_d_	344.1 ± 27.42_d_	624.14 ± 65.11_a_	439.14 ± 45.42_a_

Data in the table are mean ± standard deviation; different lowercase letters indicate significant differences between treatments for the same indicator (*P* < 0.05). CK, non-fertilized soil samples; NF, soil samples received nitrogen fertilization.

### 3.2. Soil microbial abundance and diversity

**Figure 1:**
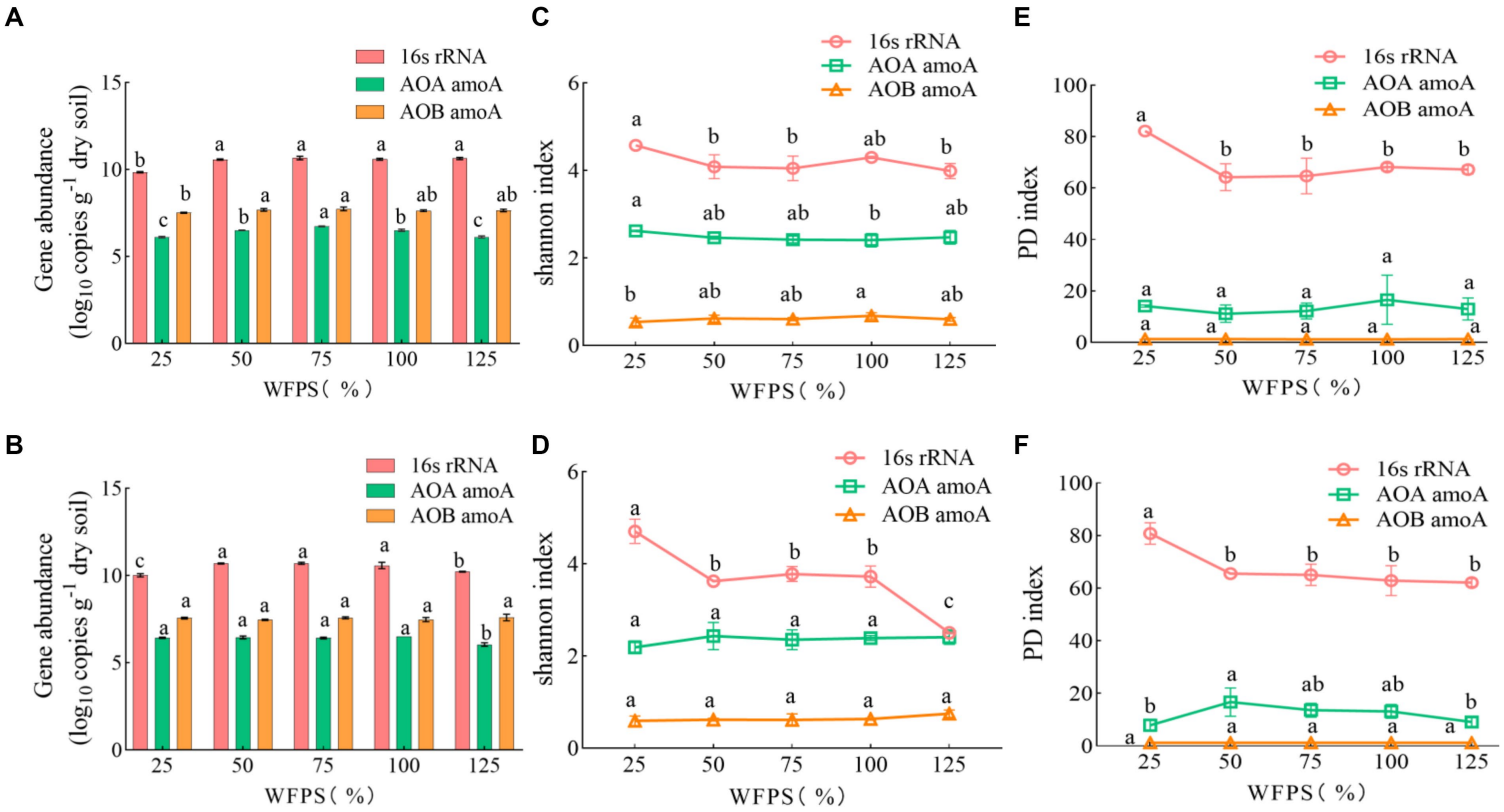
The abundance and diversity index of soil microbes under different WFPS and fertilization treatment conditions. Data are presented as mean and standard error (*n* = 3). Data labeled by different letters indicate significant differences (*p* < 0.05). **(A,C,E)** None-fertilized soil samples (CK); **(B,D,F)** Soil samples received nitrogen fertilization (NF).

Compared to diversity, the abundance of soil bacteria was more sensitive to water management, but not to N-fertilizer application (Table 2). For total bacteria, the abundance, Shannon index and PD index were affected by moisture levels. The total bacteria abundance was the lowest at 25% WFPS, while the Shannon and PD indexes were the lowest at 125% WFPS, irrespective of fertilization. For AOA and AOB communities, water management only affected their abundance, but not Shannon and PD index. AOB was always more abundant than AOA in all treatments (Figure 1). Fertilizer application had significant effect on total bacteria diversity, and decreased their Shannon index, especially under higher moisture conditions.

**Table 2:** Differences in the abundance and diversity indices of 16S rRNA, AOA and AOB affected by fertilization and water treatments.

Treatment			DF	SS	MS	F	*P*-value
Gene abundance	16S rRNA	Water	4	2.22	0.56	116.10	*P* < 0.001
		Fertilizer	1	<0.01	<0.01	0.85	*P* = 0.45
		Water × Fertilizer	4	0.33	0.08	18.43	*P* < 0.001
	AOA amoA	Water	4	0.96	0.24	119.00	*P* < 0.001
		Fertilizer	1	<0.01	0.01	1.78	*P* = 0.31
		Water × Fertilizer	4	0.31	0.08	16.71	*P* < 0.001
	AOB amoA	Water	4	0.06	0.02	4.82	*P* = 0.03
		Fertilizer	1	0.09	0.09	11.61	*P* = 0.08
		Water × Fertilizer	4	0.07	0.02	1.57	*P* = 0.28
Shannon index	16S rRNA	Water	4	5.92	1.48	32.61	*P* < 0.001
		Fertilizer	1	2.12	2.12	23.09	*P* = 0.04
		Water × Fertilizer	4	2.14	0.53	31.71	*P* < 0.001
	AOA amoA	Water	4	0.01	<0.01	0.20	*P* = 0.93
		Fertilizer	1	0.11	0.11	5.44	*P* = 0.14
		Water × Fertilizer	4	0.18	0.05	1.56	*P* = 0.28
	AOB amoA	Water	4	0.04	0.01	1.93	*P* = 0.20
		Fertilizer	1	0.01	<0.01	1.09	*P* = 0.41
		Water	4	0.03	<0.01	1.68	*P* = 0.25
PD index	16S rRNA	Fertilizer	4	1315.00	328.80	18.38	*P* < 0.001
		Water × Fertilizer	1	31.14	31.14	0.68	*P* = 0.50
		Water	4	56.13	14.03	2.05	*P* = 0.18
	AOA amoA	Fertilizer	4	71.16	17.79	0.68	*P* = 0.63
		Water × Fertilizer	1	13.46	13.46	7.16	*P* = 0.16
		Water	4	134.20	33.55	2.14	*P* = 0.17
	AOB amoA	Fertilizer	4	<0.01	<0.01	0.50	*P* = 0.74
		Water × Fertilizer	1	<0.01	<0.01	3.00	*P* = 0.23
		Water	4	<0.01	<0.01	0.50	*P* = 0.74

### 3.3. Soil bacterial community composition

**Figure 2:**
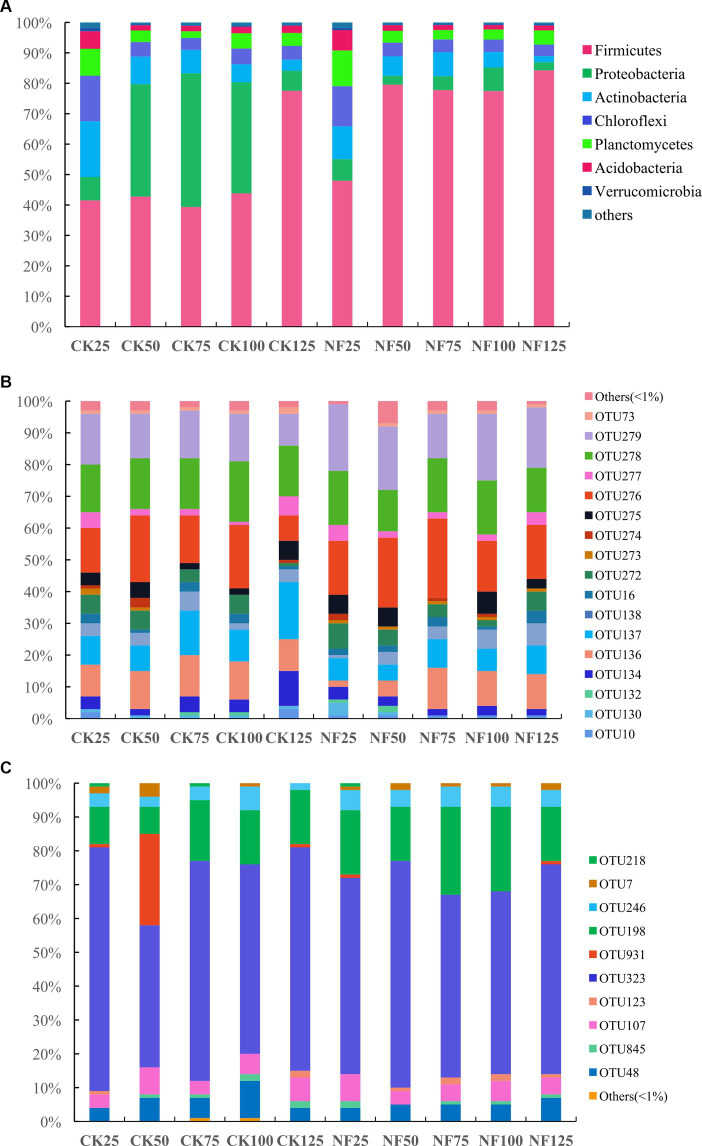
Relative abundance of soil microbes at the phylum (total bacteria) or OTU (AOA and AOB) level. CK, none-fertilized soil samples; NF, soil samples with nitrogen-fertilization; 25, 50, 75, 100, and 125 indicate the respective WFPS levels: 25%, 50%, 75%, 100%, and 125%. **(A)** total bacteria; **(B)** AOA; and **(C)** AOB.

Under 25% and 125% WFPS, the respective microbial composition on phylum level remained very similar between the CK and NF treatments. While under other WFPS conditions, fertilization significantly enhanced the relative abundance of *Firmicutes*, mainly at the expense of *Proteobacteria* abundance loss, when compared to the non-fertilized incubations (Figure 2A). The most dominant bacteria phyla were *Firmicutes*, *Proteobacteria*, *Actinobacteria*, *Chloroflexi*, *Planctomycetes*, *Acidobacteria*, and *Verrucomicrobia* (Figure 2A). The shift of relative abundance and composition of ammonia-oxidizing microorganisms at the OTU level appeared to be more responsive to water content changes (Figures 2B,C).

**Figure 3:**
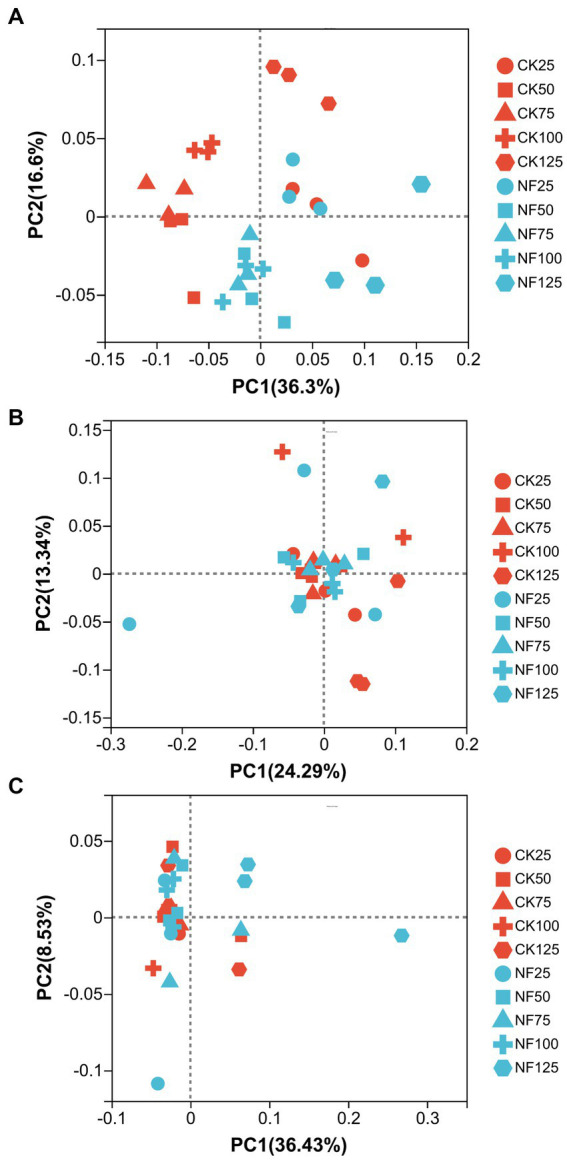
Principal coordinate analysis (PCoA) of the bacterial community structure. CK, soil samples with no N application; NF, soil samples with N-fertilization; 25, 50, 75, 100, and 125 represent WFPS level is 25%, 50%, 75%, 100%, 125%. **(A)** Total bacteria; **(B)** AOA; **(C)** AOB.

**Figure 4:**
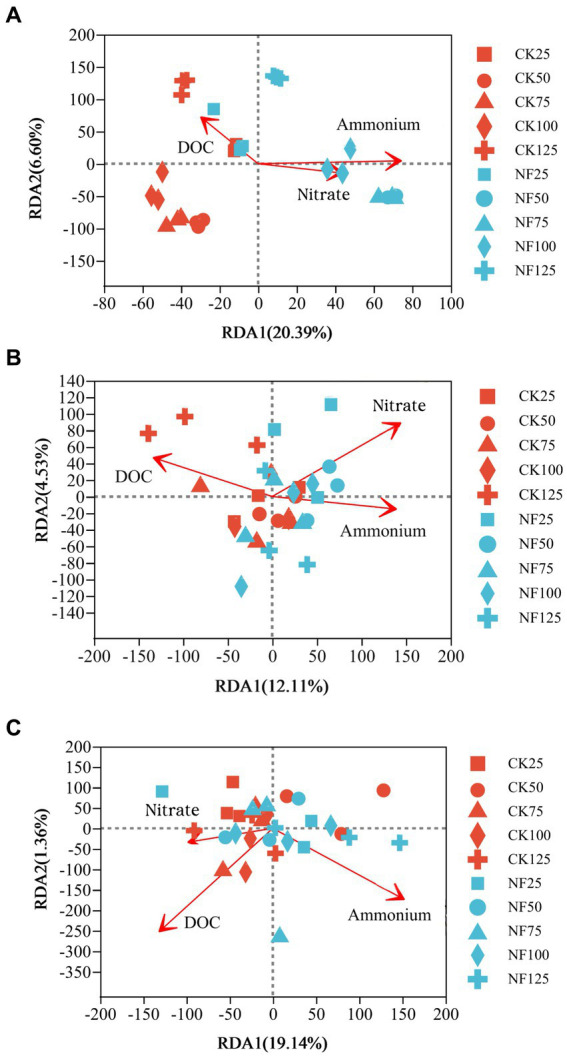
Redundancy analysis (RDA) of the effect of soil characteristics on the microbial community composition. CK, soil samples with no nitrogen application; NF, soil samples with nitrogen application; 25, 50, 75, 100, 125 represent WFPS level is 25%, 50%, 75%, 100%, 125%. **(A)** Total bacteria; **(B)** AOA; **(C)** AOB.

ANOSIM analysis indicated that both fertilization and water content had a significant effect on total bacteria community (*p* < 0.05), while their influence on AOA and AOB was less significant (Table 3). PCoA analysis showed that the total bacterial community of most incubations were separated between treatments of fertilization and non-fertilized controls (Figure 3A). The NF and CK treatments at 50%–100% WFPS were mostly grouped into respective clusters, albeit the microbial communities of both treatments at 25% and 125% WFPS were loosely distributed, indicating that “extreme” moisture conditions exerted additional influence on the bacterial community composition (Figure 3A). While, the communities of ammonia-oxidizing microorganisms could not be clearly divided between fertilization and non-fertilization treatments, nor among different WFPS levels, presumably due to the relatively slow growth rate of ammonia oxidizing microbes (Figures 3B,C). RDA analysis indicated that NO_3_^−^, NH_4_^+^ and DOC played an important role in shaping the community structure of total bacteria, AOA and AOB. Among them, the content of NO_3_^−^-N had a greater effect on AOA, and NH_4_^+^-N had the largest effect on total bacteria and AOB (Figure 4; Table 4).

**Table 3:** Intergroup similarity analysis (ANOSIM) of the fertilization and water content treatments.

ANOSIM		Statistic (R)	Pr	R^2^	*P*
16S rRNA	Fertilization	0.188	0.006	0.747	0.004
	Water	0.614	0.001	0.558	0.001
AOA	Fertilization	−0.019	0.285	0.152	0.649
	Water	0.034	0.023	0.070	0.143
AOB	Fertilization	0.031	0.31	0.155	0.235
	Water	0.123	0.003	0.133	0.015

Statistic R value, the closer to 1, the greater the difference between groups than the difference within each group; R^2^, the greater the value, the higher degree of explanation; Pr, <0.05 indicates high confidence in this test. *P*, significant difference.

**Table 4:** Redundancy analysis (RDA) of the effect of different soil characteristics on microbial community composition.

RDA analyze		RD^1^	RD^2^	R^2^	*P*
16S rRNA	Nitrite	0.998	−0.059	0.234	0.028
	Ammonium	0.998	0.064	0.696	0.001
	DOC	−0.780	0.626	0.270	0.020
AOA	Nitrite	0.856	0.518	0.470	0.001
	Ammonium	1.000	0.005	0.293	0.003
	DOC	−0.977	0.211	0.282	0.014
AOB	Nitrite	−0.599	−0.801	0.037	0.595
	Ammonium	0.903	−0.430	0.063	0.430
	DOC	0.885	−0.466	0.042	0.573

### 3.4. Network analysis

**Figure 5:**
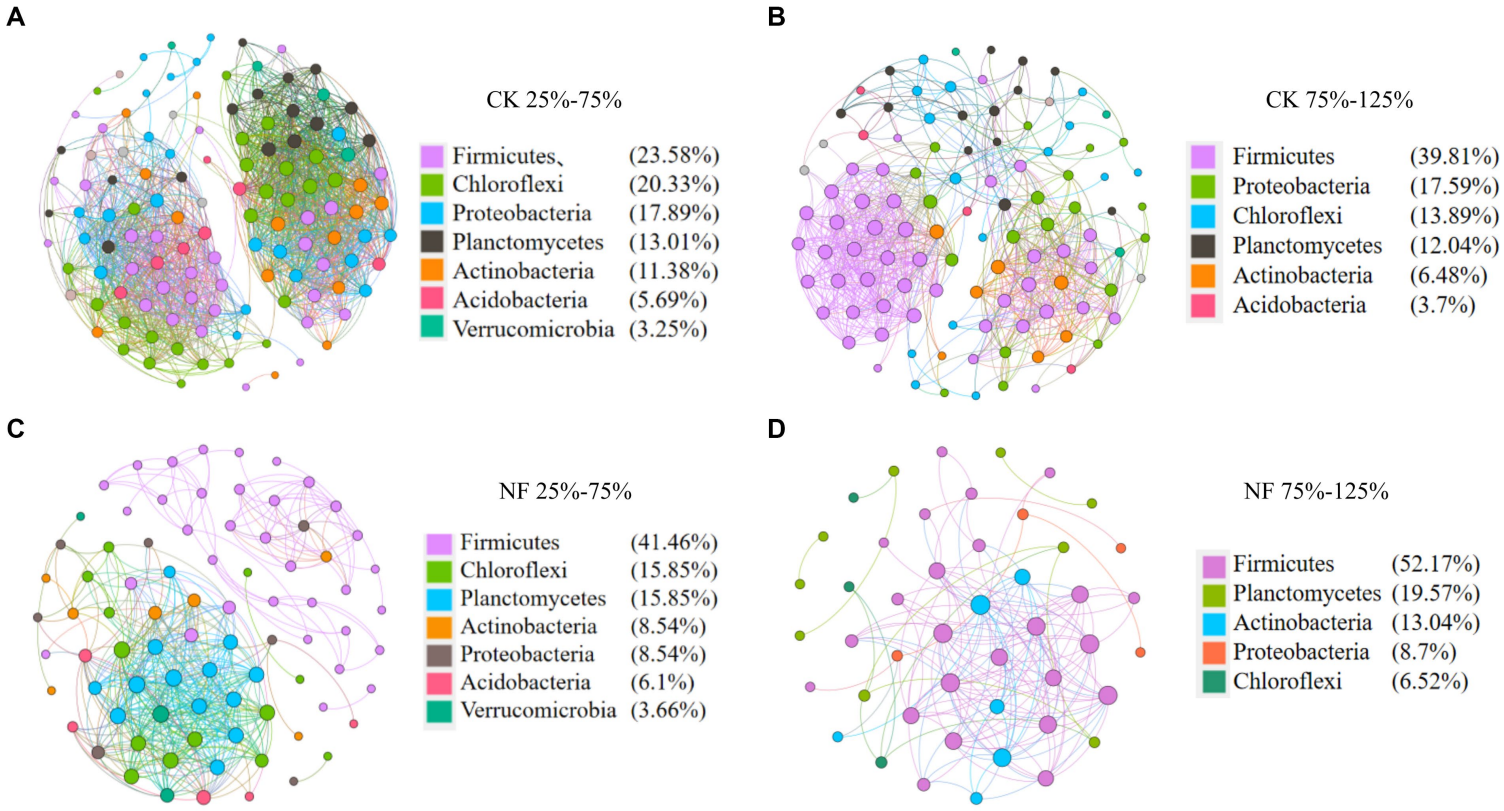
Co-occurrence analysis of total bacteria. Nodes are colored according to microbial taxonomy (phylum), edges indicate correlations among nodes. CK, none-fertilized soil samples; NF, soil samples received N-fertilization; 25, 50, 75, 100, 125 indicate respective WFPS level is 25%, 50%, 75%, 100%, 125%. **(A)** CK 25%–75%; **(B)** CK 75%–125%; **(C)** NF25%–75%; **(D)** NF 75%–125%.

Microbial network analyses showed that fertilizer application reduced the connectivity and complexity of total bacterial networks (Figure 5; Table 5). Compared to the CK treatments, the average number of nodes and edges decreased by 45.37% and 75.84%, respectively. Higher WFPS levels led to stronger decrease in the network density and clustering coefficients in NF than in CK treatment, suggesting that increasing moisture aggravated the effects of fertilization on the complexity of bacterial networks. The modularity pattern also changed with fertilization and high moisture. In the non-fertilized incubations with relatively low WFPS, two large modules were formed, each containing a number of OTUs with relatively high connectivity (Figure 5A). And all dominant phyla were represented by well-connected OTUs approximately according to their relative abundances, suggesting a rather stable microbial community was maintained. Increasing moisture or nitrogen fertilization diminished modularity, with fewer nodes forming modules. And Firmicutes, or Chloroflexi and Planctomycetes became the respective dominant taxa with well-connected OTUs (Figures 5B,C). Under simultaneous N-fertilization and high moisture, the microbial network did not form clear modules, and highly connected nodes were mostly Firmicutes.

**Table 5:** Node-level topological features of the co-occurrence network of fertilization and non-fertilized treatments grouped by moisture levels.

16S rRNA	CK 25%–75%	CK 75%-125%	NF 25%–75%	NF 75%-125%
Number of nods	123	108	82	46
Number of edges	1,634	764	458	155
Number of positive correlations	77.42%	85.08%	97.51%	81.29%
Number of negative correlations	22.58%	14.92%	2.49%	18.71%
Average degree	26.57	14.15	11.18	6.74
Network diameter	8	9	4	6
Average clustering coefficient	0.77	0.64	0.73	0.61
Average path distance	3.78	3.64	1.80	2.36
Network density	0.22	0.13	0.14	0.15

CK, soil samples with no nitrogen application; NF, soil samples with nitrogen-fertilization; WFPS level is used to divide low moisture (25%–75%) and high moisture (75%–125%) conditions.

## 4. Discussion

Soil microbial communities play an essential role in various ecological processes, including nutrient cycling, organic matter turnover, greenhouse gas emission, and soil fertility and structure maintenance. Nitrogen compounds and moisture are considered key factors shaping bacterial community in soils (Wang S. et al., 2018; Wang R. et al., 2018). However, common agricultural practices aimed at maximizing crop productivity, such as fertilization, can introduce excessive nitrogen into agroecosystems, and may have adverse ecological effects. Excessive nitrogen supply may reduce soil microbial biomass, alter microbial diversity, community structure and enzyme activity (Wang et al., 2023). Therefore, understanding how soil microbial communities respond to nitrogen fertilization and moisture fluctuations is crucial for developing sustainable agricultural practices. While several studies have examined the long-term impact of nitrogen addition on soil microbial diversity and richness in different environments, the results have been inconsistent (Fierer and Jackson, 2006; Jing et al., 2015). Investigating the immediate changes in microbial communities following short-term nitrogen fertilization and moisture variations can provide insights into the microbial response processes under long-term agricultural management.

In this study, NH_4_NO_3_ was provided as the nitrogen fertilizer, introducing both nitrate and ammonium to the NF treatments. However, after 96 h incubation, nitrate concentration in the NF incubations decreased with increasing WFPS and became undetectable under 125% WFPS. The “net NH_4_^+^” in the NF treatments also showed a decreasing trend with higher WFPS levels (Table 1), indicating a higher consumption rate of both nitrate and ammonium under increased moisture conditions. Rice paddies are well known nitrogen cycling hotspots, and denitrification and anammox are likely the main processes responsible for nitrate and ammonium consumption, especially under high WFPS levels (Zhu et al., 2011). Ammonium was available in significant concentrations, which likely supported a higher abundance of AOB compared to AOA in all incubations (Figure 1). AOA are more dominant in oligotrophic environments, while AOB tend to dominate in eutrophic habitats (Jung et al., 2022). Among the AOA OTUs, OTU276, OTU278 and OTU279 were consistently abundant in almost all incubations, while OTU323 was the most dominant AOB (Figure 2B). These OTUs had their closest cultured relatives as *Nitrososphaera viennensis* EN76 and *Nitrosospira lacus* (Tourna et al., 2011; Urakawa et al., 2015), with *amoA* sequence similarities below 80 and 86%, respectively. Hence, the AOA and AOB OTUs identified in this study were mostly uncultured, indicating a vast unexplored diversity of ammonia-oxidizing microbes in agriculture ecosystems. The abundance and composition of AOA and AOB shifted after the short-term incubation, but it remains unclear if these microbes directly contributed to the removal of ‘net NH_4_^+^’, particularly under higher WFPS conditions that were anoxic. Future studies should conduct a comprehensive analysis of the rates of different N-cycling processes, including the recently discovered oxygenic denitrification and nitrate-driven anaerobic methane oxidation (Zhu et al., 2020), to better understand their differential responses to N addition.

Soil moisture levels have been identified as a significant factor influencing soil microbial composition and activity (Shi et al., 2018). Adequate moisture levels can promote microbial growth and metabolic activities (Francioli et al., 2016; Yang et al., 2019). In our study, the microbial diversity and abundance responded differently to short-term fertilization and water management. Our results showed that total soil bacteria abundance was more responsive to water content than to fertilization (Table 2). This suggests that microbial growth was generally more strongly influenced by water content than nitrogen addition in the short-term incubation. This could be because the paddy soil was not N-limited, since there was sufficient residual ammonium present (Table 1). However, fertilization did increase bacteria diversity, likely due to the introduction of nitrate, which serves as a favorable electron acceptor for microbial respiration.

Firmicutes were the most dominant bacteria, especially in the NF treatments with higher WFPS (Figure 2). In the CK treatment with saturated moisture (125% WFPS), the relative abundance of Firmicutes (84.2%) was significantly higher than in other CK incubations (approximately 40%). This indicates a favorable response of Firmicutes to nitrogen addition and high moisture, consistent with previous findings (e.g., Supramaniam et al., 2016). Many Firmicutes are capable of forming spores, which confer high resistance to environmental stresses and enable quick response to substrate availability, making them one of the most common microbes in soils (Filippidou et al., 2016; Donhauser et al., 2021). Proteobacteria were abundant only in the CK treatments with moderate moisture levels (50%, 75%, and 100% WFPS). Interestingly, the relative abundance of Acidobacteria, Actinobacteria, Chloroflexi, and Planctomycetes was significant in both the CK and NF treatments with the lowest moisture (25% WFPS) but diminished in all other higher moisture incubations (Figure 2A). This indicates the influence of water management on soil microbial community structure. Acidobacteria and Chloroflexi members are often considered oligotrophs, and several studies have reported a decrease in their abundance with increasing concentrations of NO_3_^−^-N and NH_4_^+^-N (Zhou et al., 2015; Wang R. et al., 2018) and higher water content (Zhalnina et al., 2015; Zhou et al., 2015; Li H. et al., 2021). However, some long-term field studies have found had no significant effect of water content on soil bacterial communities (Zhang et al., 2021), possibly because there was sufficient time for the local microbial community to recover from and adapt to moisture changes (Azarbad et al., 2020). These inconsistent findings highlight the need to study the immediate responses of microbes to environmental changes.

In the present study, after a four-day incubation, it was observed that bacterial community structure, rather than total bacterial abundance, showed a stronger response to fertilization. Nitrogen fertilization has been demonstrated to be a significant factor in shaping soil microbial community (Yuan et al., 2015; Wu et al., 2021; Jin et al., 2022), probably by providing substrates and energy sources for indigenous microbes or due to nutrients imbalances resulting from a pulse input of N (Eo and Park, 2016). The bacterial abundance remained relatively stable after the 96-h incubation, which is consistent with the fact that soil bacteria generally have very slow growth rates, with a doubling time being over 100 days (Harris and Paul, 1994). Nevertheless, the shift in microbial community indicates a differential response among distinct groups of microbes. In long-term fertilizer treatments, both the structure and abundance of soil bacterial communities can also be influenced by indirect environmental changes, such as soil acidification resulting from nitrification (Zhou et al., 2015; Wang et al., 2016; Lin et al., 2020). However, some studies suggest that soil parent material plays a key role in shaping agricultural soil bacterial communities from a long-term perspective (Sheng et al., 2023).

The number of nodes, edges and average clustering coefficients decreased in the microbial networks of the NF treatment and higher moisture incubations, and the interaction pattern among soil microbes also shifted (Table 5). In the non-fertilized treatment, increasing moisture reduced the ratio of negative interactions, which represent microbial competition and are considered important for stabilizing microbial communities and their ecological services (van der Heijden et al., 2010; Hoek et al., 2016). These results indicated that both fertilization and higher water content rapidly reduced the complexity and stability of soil bacterial communities. Based on the degree of each node, key taxa identified in the microbial network mostly belonged to Firmicutes, Proteobacteria, Actinobacteria, Chloroflexi and Planctomycetes, indicating their crucial role in paddy soils under fertilization and water management. In recent years, the construction of microbial network has become increasingly popular for high-throughput sequencing data analysis and been helpful in uncovering interactions from complex datasets (Deng et al., 2012; Faust and Raes, 2012). However, it should be noted that the inference of biotic interactions from the cooccurrence or absence of sequences may not always reliable, and conclusions should be drawn with caution (Faust, 2021).

## 5. Conclusion

The composition and abundance of soil bacteria and ammonia-oxidizing microorganisms in paddy fields demonstrated varying degrees of response to fertilization and moisture, with a higher sensitivity to changes in water content levels. The impact of fertilization on the stability and complexity of bacteria networks was evident and slightly exacerbated by elevated moisture. Changes in the total bacteria and AOB community showed significant correlations with NH_4_^+^-N content, while NO_3_^−^-N content played a crucial role in driving changes in the AOA community structure. Studying the initial changes in microbial community composition and structure in response to water and fertilizer applications can provide valuable insights into how soil microbes adapt to long-term agricultural practices, thus aiding in the development of more sustainable agricultural approaches.

## Data availability statement

The original contributions presented in the study are included in the article/supplementary material, further inquiries can be directed to the corresponding authors.

## Author contributions

LH, HQ, JW, DY, and LZ carried out the experiments. HQ, LH, BZ, and JG did sequencing and data analysis. LH, HQ, JG, and BZ wrote the manuscript. All authors contributed to the article and approved the submitted version.

## Funding

The research was supported by the National Key Research and Development Program of China (2021YFD1901203), the National Natural Science Foundation of China (project no. 42177104), and the National Ecosystem Science Data Center (no. NESDC20210204).

## Conflict of interest

The authors declare that the research was conducted in the absence of any commercial or financial relationships that could be construed as a potential conflict of interest.

## Publisher’s note

All claims expressed in this article are solely those of the authors and do not necessarily represent those of their affiliated organizations, or those of the publisher, the editors and the reviewers. Any product that may be evaluated in this article, or claim that may be made by its manufacturer, is not guaranteed or endorsed by the publisher.
